# D-allose Inhibits TLR4/PI3K/AKT Signaling to Attenuate Neuroinflammation and Neuronal Apoptosis by Inhibiting Gal-3 Following Ischemic Stroke

**DOI:** 10.1186/s12575-023-00224-z

**Published:** 2023-11-28

**Authors:** Yaowen Luo, Junkai Cheng, Yihao Fu, Min Zhang, Maorong Gou, Juan Li, Xiaobing Li, Jing Bai, Yuefei Zhou, Lei Zhang, Dakuan Gao

**Affiliations:** 1 Department of Neurosurgery, Xijing Hospital, Air Force Medical University, Changle West Road NO.127, Xi’an, China; 2 Department of Neurology, Xijing Hospital, Air Force Medical University, Changle West Road 127, Xi’an, China

**Keywords:** D-allose, Gal-3, TLR4 Signaling, Neuroinflammation, Ischemic Stroke, Neurological Dysfunction

## Abstract

**Background:**

Ischemic stroke (IS) occurs when a blood vessel supplying the brain becomes obstructed, resulting in cerebral ischemia. This type of stroke accounts for approximately 87% of all strokes. Globally, IS leads to high mortality and poor prognosis and is associated with neuroinflammation and neuronal apoptosis. D-allose is a bio-substrate of glucose that is widely expressed in many plants. Our previous study showed that D-allose exerted neuroprotective effects against acute cerebral ischemic/reperfusion (I/R) injury by reducing neuroinflammation. Here, we aimed to clarify the beneficial effects D-allose in suppressing IS-induced neuroinflammation damage, cytotoxicity, neuronal apoptosis and neurological deficits and the underlying mechanism in vitro and in vivo.

**Methods:**

In vivo, an I/R model was induced by middle cerebral artery occlusion and reperfusion (MCAO/R) in C57BL/6 N mice, and D-allose was given by intraperitoneal injection within 5 min after reperfusion. In vitro, mouse hippocampal neuronal cells (HT-22) with oxygen–glucose deprivation and reperfusion (OGD/R) were established as a cell model of IS. Neurological scores, some cytokines, cytotoxicity and apoptosis in the brain and cell lines were measured. Moreover, Gal-3 short hairpin RNAs, lentiviruses and adeno-associated viruses were used to modulate Gal-3 expression in neurons in vitro and in vivo to reveal the molecular mechanism.

**Results:**

D-allose alleviated cytotoxicity, including cell viability, LDH release and apoptosis, in HT-22 cells after OGD/R, which also alleviated brain injury, as indicated by lesion volume, brain edema, neuronal apoptosis, and neurological functional deficits, in a mouse model of I/R. Moreover, D-allose decreased the release of inflammatory factors, such as IL-1β, IL-6 and TNF-α. Furthermore, the expression of Gal-3 was increased by I/R in wild-type mice and HT-22 cells, and this factor further bound to TLR4, as confirmed by three-dimensional structure prediction and Co-IP. Silencing the Gal-3 gene with shRNAs decreased the activation of TLR4 signaling and alleviated IS-induced neuroinflammation, apoptosis and brain injury. Importantly, the loss of Gal-3 enhanced the D-allose-mediated protection against I/R-induced HT-22 cell injury, inflammatory insults and apoptosis, whereas activation of TLR4 by the selective agonist LPS increased the degree of neuronal injury and abolished the protective effects of D-allose.

**Conclusions:**

In summary, D-allose plays a crucial role in inhibiting inflammation after IS by suppressing Gal-3/TLR4/PI3K/AKT signaling pathway in vitro and in vivo.

## Introduction

Globally, ischemic stroke (IS) is currently one of the leading causes of death and long-term disability, and there is a shortage of effective treatments despite numerous clinical trials because the pathophysiology of the various cells in injured brain tissue is not yet well understood [1–3]. Many studies have indicated that the inflammatory response has a direct negative effect on ischemic neurons, which mediate brain function, leading to the collapse of organelle structure and the release of proapoptotic proteins, ultimately causing cerebral apoptosis and necrosis [4–7]. Currently, reperfusion of the infarct is a major therapeutic goal. Intravenous thrombolysis is one of the few highly effective treatments for acute IS, but it does not consider the ischemic cascade that takes place following the onset of IS, including oxidative injury, inflammation damage and cell death [8]. Many experimental and clinical studies have been performed to discover drugs that can mitigate neuronal cell injury and neurodegeneration, as well as cell death after the ischemic cascade [9]. However, scientists and researchers have not yet found a drug with a multifunctional and definitive effect. Therefore, protecting neurons from inflammatory damage and apoptosis could be a rational and efficient measure to ameliorate neurological dysfunction in IS patients.

D-allose (PubChem CID: 439,507), a C-3 epimer of D-glucose that was originally isolated from some plants, is an important rare monosaccharide that is safe for consumption by humans [10]. Accumulating evidence has shown that D-allose inhibits the production of reactive oxygen species (ROS) in inflamed leukocytes [11], activates programmed apoptosis in tumor cells [12], and exerts an anti-inflammatory effect to protect the kidney [13], liver [14] and retina [15] from ischemia/reperfusion(I/R) injury. Our previous study examined the anti-inflammatory effects of D-allose on a model of focal cerebral I/R injury in rats [16]. However, whatever the mechanism of the neuroprotective effect of D-allose after IS involves simultaneously reducing neuroinflammation-induced damage and neuronal apoptosis remains unclear.

Galectin-3 (Gal-3), an important member of the β-galactoside- binding lectin family, was first identified in murine immune cells and later found in microglia, astrocytes and neurons and was shown to be a novel inflammatory factor involved in inflammation, oxidative stress, cellular proliferation, apoptosis and pyroptosis [17–21]. The effect of Gal-3 on neuroinflammation in neurodegeneration and central nervous system (CNS) injury is controversial [22]. Some studies have shown that deletion of the Galectin-3 gene attenuates injury by decreasing the expression levels of inflammatory cytokines in C57BL/6 mice during autoimmune encephalomyelitis progression [23]. Other studies indicated that Gal-3 deficiency in the microglia and astrocytes of C57BL/6 mice enhanced the inflammatory response in Wallerian degeneration (WD) [24]. Nevertheless, the function of Gal-3 in apoptosis is somewhat controversial. Wesley et al. reported that Gal-3 was involved in neurovascular protection and functional recovery after IS through the upregulation of cerebral blood vessel density and downregulation of neuronal apoptotic death [25, 26]. In contrast, Fukumori T et al. showed that tumor cell-secreted Gal-3 induced T-cells apoptosis, which in turn enhanced the antiapoptotic effect of Gal-3 on tumor cells [27]. In summary, the effects of Gal-3 on inflammatory damage-induced neuronal apoptosis after IS are largely unknown.

Secondary neuroinflammation can occur after acute ischemic stroke. Immune-mediated proinflammatory signals rapidly activate various types of inflammatory cells, causing them to infiltrate ischemic areas and exacerbate brain damage [28]. Among the inflammatory-related proteins and signaling pathways, Toll-like receptor 4 (TLR4) and TLR4-dependent PI3K/AKT signaling are crucial inflammatory pathways and are related to the pathogenesis and development of many neuroinflammatory diseases and are involved in protecting the brain from I/R-induced injury [29–34]. Mietto BS et al. observed that a lack of Gal-3 increased the RNA and protein levels of proinflammatory cytokines, as well as TLR2 and TLR4, which ultimately contributed to the prognosis of WD [24]. Conversely, Liu et al. recently reported that TD139, a specific inhibitor of Gal-3, alleviated TLR4 and MyD88 activation in microglia in autoimmune uveitis [35]. Therefore, the relationship between the TLR4/PI3K/AKT signaling axis and Gal-3 in inflammatory damage to neurons induced by IS remains unknown. Moreover, similar to TD139, whether D-allose plays an anti-inflammatory role by inhibiting the expression of Gal-3 and the TLR4 signaling pathway in IS also remains unclear.

The aims of this study were (1) to determine whether D-allose pretreatment alleviated inflammatory damage and ischemic neuronal apoptosis induced by brain I/R injury and, if so, (2) to evaluate the underlying mechanisms of Gal-3 downregulation via TLR4/ PI3K/AKT signaling in mediating the protective effects of D-allose.

## Materials and Methods

### Mice and the Cerebral-Ischemia Reperfusion Model

One hundred and twenty C57BL/6 N male mice (aged 8–12 weeks with a body weight of 20–30 g) were housed under controlled environmental conditions in standard cages with a consistent temperature of 22–25 ℃, relative humidity of 65%, and a 12 h light/dark cycle. The mice were provided unrestricted access to food and water. All experimental protocols and animal handling procedures in this study were performed in accordance with the National Institutes of Health (NIH) guidelines for the use of experimental animals, and the study received approval from the Institutional Animal Care and Use Committee of the Air Force Medical University (No. IACUC-20,220,122).

As referred to in a prior investigation [36], the middle cerebral artery occlusion model was successfully established in our study. Cerebral ischemia was induced by middle cerebral artery occlusion (MCAO). General anesthesia was induced with 5% isoflurane (RWD, Shenzhen, China) in a mixture of 70% nitrous oxide and 30% oxygen and maintained with 2% isoflurane delivered via facemask during spontaneous respiration. Rectal temperature was closely monitored and maintained within the range of 36.5–37.5 °C throughout the surgery with the aid of warming blankets and lamps until the animal regained consciousness. Briefly, a midline incision was made in the jugular skin to expose the unilateral common carotid artery (CCA), external carotid artery (ECA), and internal carotid artery (ICA). Subsequently, the CCA and ECA were ligated while the ICA was clipped using microvascular aneurysm clips. Following arteriotomy in the ECA, a soft silicone-coated surgical nylon monofilament suture (0.12 mm diameter; 3.0 cm length, RWD Life Science, China) was gently inserted into the ICA via the ECA to occlude the middle cerebral artery (MCA), approximately 12.0 mm distal to carotid bifurcation. Reperfusion was allowed by removing the filaments after a 2-hour period. Neurological deficit scores were evaluated 24 and 48 h after MCAO, followed by decapitation to obtain the brains for subsequent experiments. In the Sham group, the mice underwent the same procedure without the insertion of the nylon monofilament to occlude the middle cerebral artery (MCA). Intraperitoneal administration of 0.4 mg/g D-allose, which was dissolved in normal saline, was administered within 5 min after reperfusion. The doses used in this study were determined based on our preliminary experiments and previous research [37].

### Cell Culture and the Oxygen–Glucose Deprivation and Reperfusion Model

Mouse hippocampal HT-22 cells were obtained from ATCC (USA) and cultured in Dulbecco’s modified Eagle’s medium (DMEM) supplemented with 1% penicillin/streptomycin (Gibco, USA) and 10% fetal bovine serum (Sijiqing, China) at 37 ℃ and 5% CO_2_ in a Thermo Fisher incubator. The culture medium was refreshed every 48 h, and subculturing was performed when cell confluence reached approximately 80–90%. Only logarithmically growing cells were used for subsequent experiments.

To induce oxygen-glucose deprivation (OGD), the medium was removed, and the cells were rinsed three times with phosphate-buffered saline (PBS). The cultured cells were placed in a dedicated chamber containing CO_2_/N_2_ (5%/95%) at 37 °C, and DMEM was replaced with glucose-free DMEM, which was preprocessed with N_2_/ CO_2_ (95%/5%) to remove other gasses. According to the experimental design, the cells were removed from the anoxic chamber. Then, glucose-free DMEM was replaced with neurobasal medium, and the cells were cultured under normoxic conditions CO_2_/O_2_/N_2_ (5%/21%/74%) for 24 h of reoxygenation to induce reperfusion injury. Control cells were cultured under normoxic conditions as part of routine culture procedures. During reoxygenation, 1.8 mg/ml D-allose, which was dissolved in PBS, was added to the medium.

### Neurological Score Evaluation

Mice that died shortly after MCAO/R were excluded. Evaluations were performed 24 h after reperfusion, and immediately prior to brain tissue sampling. The Modified Neurological Severity Score (mNSS) [38, 39] was used to assess the severity of nerve injury in the experimental animals. The degree of neurological deficits was graded on a scale from 0 to 18, with higher scores indicating greater damage.

### Infraction Volume Ratio and Brain Edema Measurement

Excessive administration of pentobarbital sodium was used for euthanasia following exposure to ischemic conditions. The brains were sliced into 2 mm coronal sections with an even thickness. Subsequently, the sections were stained using a 2% solution of 2,3,5-triphenyl tetrazolium chloride (TTC) (Solarbio, Beijing, China) at 37 °C for 20 min [40]. The stained brain sections were fixed in 2% paraformaldehyde and imaged, after which the infarct volume (white) percentage was analyzed using ImageJ software (National Institutes of Health, USA). In brief, the infarct volume was calculated by multiplying the total area of the infarction by the section thickness (2 mm). The ratio of the infarct volume to total brain volume is indicative of cerebral infarction.

Brain edema was evaluated by measuring brain water content using the dry-wet weight method [41]. In brief, following reperfusion, the mice were administered an overdose of anesthesia, and their brains were promptly harvested. The brains were then dissected along the midsagittal plane to separate the ischemic hemisphere from the normal hemisphere. Each hemisphere was immediately weighed, and the samples were dried in an oven at 100 °C until a constant weight was achieved to determine wet weight and dry weight. The brain water content was calculated as [(wet weight - dry weight)/wet weight] × 100%.

### TUNEL Staining

Terminal Deoxynucleotidyl Transferase (TdT)-mediated dUTP Nick-End Labeling (TUNEL) (Beyotime, Shanghai, China) staining was used to visualize neuronal apoptosis [42]. In brief, cells and brain sections were fixed with freshly prepared 4% paraformaldehyde for 20 min at room temperature and washed twice with PBS. Then, to inhibit endogenous peroxidase activity in the brain, the sections were incubated with a methanol solution containing 0.2% H2O2 for 30 min. Subsequently, the samples were treated with 50 µl of TUNEL reaction mixture and incubated in a dark and humidified atmosphere at 37 ℃ for 60 min. The nuclei were then stained with 4′,6-diamidino-2-phenylindole (DAPI). The images were acquired using a fluorescence microscope (Olympus, Tokyo, Japan). The results are expressed as the apoptosis index, which was calculated by dividing the number of TUNEL-positive cells by the total number of cells in five randomly selected fields for each condition and multiplying by 100%.

### Immunohistochemistry and Immunofluorescence Analysis

The mice were anesthetized and transcardially perfused with ice-cold heparin saline, followed immediately by a 4% formaldehyde solution. The brain was subsequently removed and immersed in a 4% formaldehyde solution, as well as 15% and 30% sucrose solutions, overnight at 4 ℃. Afterward, the tissues were sectioned into coronal slices with a thickness of 16 μm using a cryostat microtome (Leica, Wetzlar, Germany). HT-22 cells were cultured on cell slides in 6-well plates and fixed in 4% paraformaldehyde for 15 min at room temperature. The brain sections were washed in PBS for 30 min, and the cells were rinsed with PBS. The samples were permeabilized with 0.1% Triton X-100 in PBS for 10 min and blocked with 5% bovine serum albumin (BSA) (Solarbio, Beijing, China) for 30 min at room temperature before being rinsed three times in PBS. For antibody staining, the sections were incubated overnight at 4 ℃ with primary antibodies against galectin-3 (1:250, Abcam, USA) and NeuN (1:200, Cell Signaling Technology, USA). After the samples were washed in PBS for 10 min, they were incubated with fluorescent secondary antibodies (1:500, Invitrogen, USA) for 1 h at 37 ℃. Finally, the slices were overlaid with mounting medium containing DAPI to counterstain nuclei. The immunofluorescence images were captured using an Olympus fluorescence microscope (Tokyo, Japan). Five random regions of each sample were imaged and analyzed using ImageJ software (National Institutes of Health, USA).

### Analysis of Cell Viability

Cell viability was assessed using the Cell Counting Kit-8 (CCK-8) assay (Beyotime, Shanghai, China) according to the manufacturer’s instructions. HT-22 cells were seeded in 96-well plates under normoxic conditions until they adhered. The cells were subjected to oxygen-glucose deprivation (OGD) the indicated time, followed by the re-establishment of normal culture conditions for an additional 24 h with or without therapeutic intervention. The optical density (OD) value at 450 nm in each well was quantified using a microplate reader (Bio-Rad, Hercules, California, USA).

### Lactate Dehydrogenase (LDH) Release Assay

The release of cytoplasmic LDH is indicative of compromised cell membrane integrity, which signifies cell death [43]. The LDH content in the cell supernatants was quantified using an LDH kit according to the instructions provided by the manufacturer. The medium was added to each well of a 24-well plate that was placed within an optically clear 96-well plate for a subsequent coupled enzymatic reaction. The LDH Reaction Mix was added to each well, mixed thoroughly, and incubated at 37 ℃ for 30 min to generate a brownish-red product. The absorbance of the product was measured spectrophotometrically at 450 nm using a microplate reader (Bio-Rad, Hercules, California, USA).

### RT-PCR

Total RNA was extracted from mouse brains and cells using TRIzol reagent (Sigma Aldrich, USA). The RNA concentration was quantified using spectrophotometry (Thermo Fisher Scientific, Waltham, USA). Reverse Transcription Master Mix (Takara, Tokyo, Japan) was used to synthesize cDNA by reverse transcription at 42 ℃ for 15 min followed by a denaturation step at 85 ℃ for 5 s. mRNA expression was analyzed using SYBR Premix Ex Taq TM II (Takara, Tokyo, Japan) and synthetic primers on a thermocycler (Bio-Rad, Hercules, California, USA). Relative mRNA expression levels were calculated and quantified with the 2 − ΔΔCt method after normalization to reference GAPDH expression [44]. The primers used for quantitative real-time PCR in this study are listed in Table 1.

**Table 1:** Primer sequences of GAPDH and Lgals 3 genes

Gene	Primer sequence(5′-3′)	Base
Lgals3	F: CCCTTTGAGAGTGGCAAACCA R: CATCGTTGACCGCAACCTT	21 19
Gapdh	F: GGTGAAGGTCGGTGTGAACG R: CTCGCTCCTGGAAGATGGTG	20 20

### Western Blot Analysis

Radioimmunoprecipitation assay (RIPA) lysis and extraction buffer containing 1% Phenylmethanesulfonyl fluoride (PMSF) was used to extract total protein from damaged tissue and cells. Protein quantification was performed using a BCA kit (Beyotime, Shanghai, China). Equal amounts of protein (30–50 µg) from each sample were separated using an 8–12% sodium dodecyl sulfate SDS-PAGE gel and subsequently transferred onto polyvinylidene difluoride (PVDF) membranes. The membranes were then blocked with a 5% skim milk solution in TBST for one hour at room temperature, followed by overnight incubation with specific primary antibodies at 4 °C on a shaker. Subsequently, the membranes were subjected to three washes with TBST and then probed with HRP-conjugated secondary antibodies diluted in TBST for 1 h at room temperature while being constantly agitated. The protein bands were visualized using an ECL substrate (Thermo Fisher Scientific, Waltham, USA) and imaged by a detection system (Bio-Rad, Hercules, California, USA). The optical densities of the bands were scanned and quantified using image-analysis software (ImageJ software, National Institutes of Health, USA), and β-actin served as an internal control.

### 3D Structure Prediction

The structure of murine Galectin-3 used in this study was obtained from the PDB database (accession number 7CXB), and murine TLR4 protein had the accession number 2Z64. First, protonation processing under neutral conditions (pH = 7) was performed using the H + + 3 online server. Subsequently, UCSF Chimera software was used to eliminate heteroatoms and water molecules from the crystal structure, and retain only the protein structure with Amber14SB charge allocation. Next, the protein-protein docking tool HDOCK was used for molecular docking, and the experience-based iterative scoring function ITScorePP was used for molecular docking and configuration scoring. A negative score indicates successful binding of molecules, and greater absolute values indicate stronger binding abilities. The maximum number of output configurations for docking was set at 100, and only the top 10 configurations were scored and analyzed using confidence scores to determine reliability. If the value exceeded 0.7, it signified a reliable docking score and a high likelihood of molecular binding. Conversely, if the value fell below 0.7, it indicated a low reliability of the docking score and a decreased possibility of binding. The docking configuration with the highest docking score and confidence score was chosen for subsequent analysis. PyMOL2.04 was used for 3D mapping analysis, and Maestro was used for 2D interaction analysis and statistical determination of the interaction type, distance, and quantity.

### Coimmunoprecipitation (CO-IP)

#### Coi

mmunoprecipitation (Co-IP) assays were performed in accordance with established protocols [45]. The cells were scraped in NP-40 immunoprecipitation lysis buffer (Beyotime China) containing phenylmethanesulfonyl fluoride (PMSF) and protease inhibitors using a cell scraper. After being lysed for 30 min on ice, the cells were centrifuged at 12,000 rpm for 30 min at 4 ℃. The resulting supernatant was collected and subsequently incubated with primary antibodies (TLR4 and Gal-3) or isotype immunoglobulin G (IgG). (#3900, Cell Signaling Technology, Shanghai, P.R. China), with gentle rocking for 2 h at 4 °C. Subsequently, 35 µL of protein A/G beads (#sc-2003, Santa Cruz, Shanghai, China) were added to each immunoprecipitation mixture and allowed to stand overnight at 4 °C. On the following day, the mixtures were subjected to five rounds of cold 1×Co-IP buffer washes, followed by denaturation of the bound protein with 1× sample buffer. The resulting supernatants were collected and used for SDS-PAGE and Western blot analysis.

### Enzyme‑Linked Immunosorbent Assay (ELISA)

The brain tissues were thoroughly ground and the supernatant of the homogenate was collected after centrifugation. The cell culture supernatants were also collected. IL-1β, IL-6, and TNF-α levels were detected using a commercial ELISA kit (Nanjing Jiancheng Bioengineering Institute, China).

### shRNA Transfection

The Gal-3 knockdown lentiviruses (Hanheng China) were transiently transfected into the cells using a transfection reagent (Beyotime China) according to the manufacturer’s instructions. Transfection efficiency was evaluated by harvesting the cells after 3 days. The sequences of the Gal-3 and control shRNAs are shown in Table 2. Stable HT-22 cell lines with Gal-3 knockdown were established through lentiviral infection and subsequent selection with 5 µg/ml puromycin (Sigma, China) for approximately one week. Finally, the stability of the generated cell lines was confirmed by Western blot analysis and qPCR.

The mice were anesthetized with an intraperitoneal injection of chloral hydrate (400 mg/kg, Sigma) and secured onto a stereotactic frame (RWD, Shenzhen, China). Rectal temperature was maintained at 37.5 °C using a heated blanket. A midline scalp incision was performed to fully expose the bregma and lambda. For intracortical injections, a borehole was created on the left side using a high-speed drill at coordinates relative to the bregma (X = 1.0 mm, Y = 0.80 mm). The syringe was connected to a microinjection pump, the needle was inserted into the brain through the burr hole (Z = 1.5 mm from the bone surface), and 1 µl of Gal-3 knockdown adeno-associated virus (AAV) (Hanheng China) was infused [46]. The Gal-3 and control shRNA sequences are shown in Table 2. Following the surgery, the cranial defect was sealed using bone wax, and the incision was closed with sutures.

**Table 2:** Primer sequences of LV and AAV

Virus	Designation	Sequence
LV	Con	Top: GAT CCG TTC TCC GAA CGT GTC ACG TAA TTC AAG AGA TTA CGT GAC ACG TTC GGA GAA TTT TTT C
		Bottom: AAT TGA AAA AAT TCT CCG AAC GTG TCA CGT AAT CTC TTG AAT TAC GTG ACA CGT TCG GAG AAC G
	Aim	Top: GAT CCG CCC AAC GCA AAC AGG ATT GTT CTC GAG AAC AAT CCT GTT TGC GTT GGG TTT TTT G
		Bottom: AAT TCA AAA AAC CCA ACG CAA ACA GGA TTG TTC TCG AGA ACA ATC CTG TTT GCG TTG GGC G
AAV	Con	Top: GAT CCG TTC TCC GAA CGT GTC ACG TAA TTC AAG AGA TTA CGT GAC ACG TTC GGA GA
		Bottom: AAT TGA AAA AAT TCT CCG AAC GTG TCA CGT AAT CTC TGA ATT ACG TGA CAC GTT CGG AGA ACG
	Aim	Top: AAT TCG CCC GCT TCA ATG AGA ACA ACA CTC GAG TGT TGT TCT CAT TGA AGC GGG TTT TTT G
		Bottom: GAT CCA AAA AAC CCG CTT CAA TGA GAA CAA CAC TCG AGT GTT GTT CTC ATT GAA GCG GGC G

### Replicates

For in vivo molecular biology experiments, three technical replicates of each mouse were performed, the data were averaged and analyzed, and the mean values were obtained from three mice per group (*n* = 3/group) and used for intergroup comparisons. In vitro, cells in three wells were examined to obtain the mean counts, and the results were measured from three independent biological replicates in each experiment.

For pathology experiments, sections from three mice per group and cells in three wells were observed under a microscope, three randomly selected fields of view from each section were used to quantify the assay metrics and the data were averaged and analyzed. The three mean values obtained for each group (*n* = 3/group) were used for comparisons between groups.

### Statistical Analysis

PASS 15.0 software was used for power analysis. One-way ANOVA was used to calculate the appropriate sample size with the following statistical parameters: a power value of 0.9 and an alpha value of 0.05.

The experiments were repeated at least three times, and the data are presented as the mean ± standard deviation. Statistical analysis was performed using GraphPad Prism 9.0 (GraphPad Software, La Jolla, CA, USA). The data among multiple groups were compared with one-way ANOVA followed by Dunnett’s test. *P*>0.05 was considered to be statistically significant.

## Results

### D-allose Protects the Brain from MCAO/R-induced Injury in vivo

**Fig. 1:**
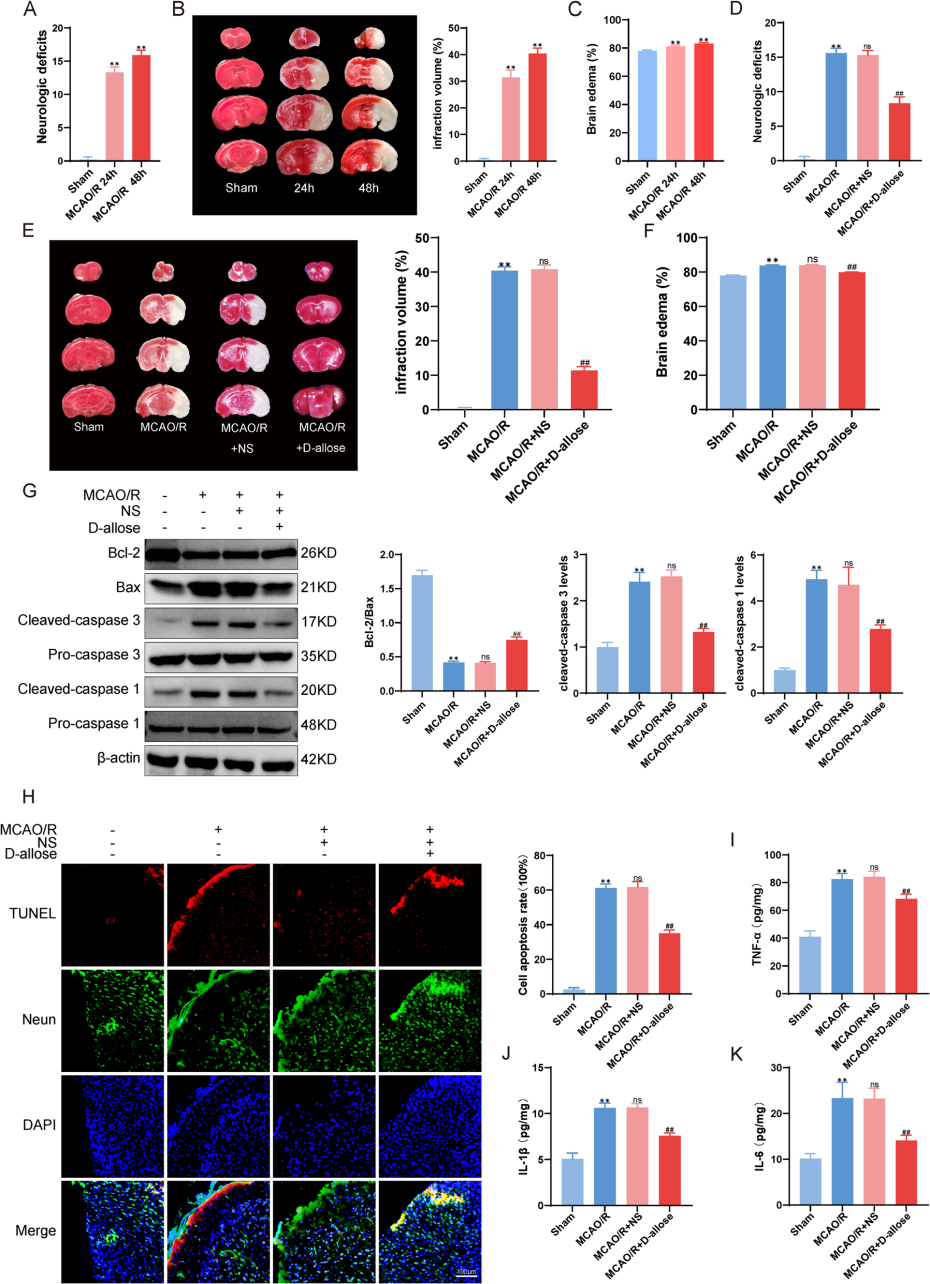
The neuroprotective effects of D-allose.  **A** Neurological function was assessed using the mNSS scale 24 and 48 h after ischemia-reperfusion. **B** Cerebral infarct volumes were measured using TTC staining 24 and 48 h after reperfusion. **C** Brain edema was measured 24 and 48 h after reperfusion. **D** Assessment of neurological function after the treatment. **E** D-allose was administered by intraperitoneal injection within 5 min after reperfusion. TTC staining was performed to evaluate the infarct volumes. **F** Brain edema was measured after the treatment. **G** Western blot analysis of apoptosis-related molecules. **H** TUNEL assay of neuronal apoptosis in mice after MCAO.  The levels of TNF-α (**I**), IL-1β (**J**) and IL-6 (**K**) in cerebral tissue were detected by ELISA kits. ** *P*<0.01 compared with the Sham group; ^ns^*P*>0.05, ^##^*P*<0.01 compared with the MCAO group. The data were analyzed using one-way ANOVA and Dunnett’s test was used for post hoc analysis. Data were from three independent experiments

In this study, we used the MCAO/R model in C57BL/6 N male mice (aged 8–12 weeks), which can mimic human IS, to assess the neuroprotective effect of D-allose. Compared with mice in the Sham group, mice in the MCAO/R group showed strong neurological deficits, obvious infarct volumes and brain edema (*P* < 0.01, Fig. 1A-C), suggesting that serious brain injury was induced by MCAO/R model. As showen in Fig. 1D-F, in the MCAO/R and MCAO/R + NS groups, transient MCAO/R induced an evident and defined infarct, brain edema and neurological deficits. Intraperitoneal injection of 0.4 mg/g D-allose which within 5 min after reperfusion significantly reduced the brain infarct volume by 30% and brain edema by 4% and improved the mNSS score by 45% at 48 h after MCAO/R. Moreover, the presence of D-allose significantly decreased the levels of the apoptosis markers Bax, cleaved Caspase 3 and cleaved Caspase1, as well as the proportion of apoptosis-positive neurons in the ipsilateral cortex, which was examined by Western blotting and TUNEL staining (*P* < 0.01, Fig. 1G-H). Moreover, as shown in Fig. 1I-K, the level of the classic inflammatory cytokines IL-1β, IL-6 and TNF-α were markedly increased in mice subjected to MCAO/R. D-allose treatment significantly reduced the levels of IL-1β, IL-6 and TNF-α compared with those in the MCAO/R group (*P* < 0.01). Taken together, these results suggested that D-allose could protect the brain from MCAO/R-induced apoptosis and inflammation and suggests the potential for clinical trials of IS treatment with D-allose in the future.

### D-allose Protects HT-22 Cells from OGD/R-induced Cytotoxicity in vitro

**Fig. 2:**
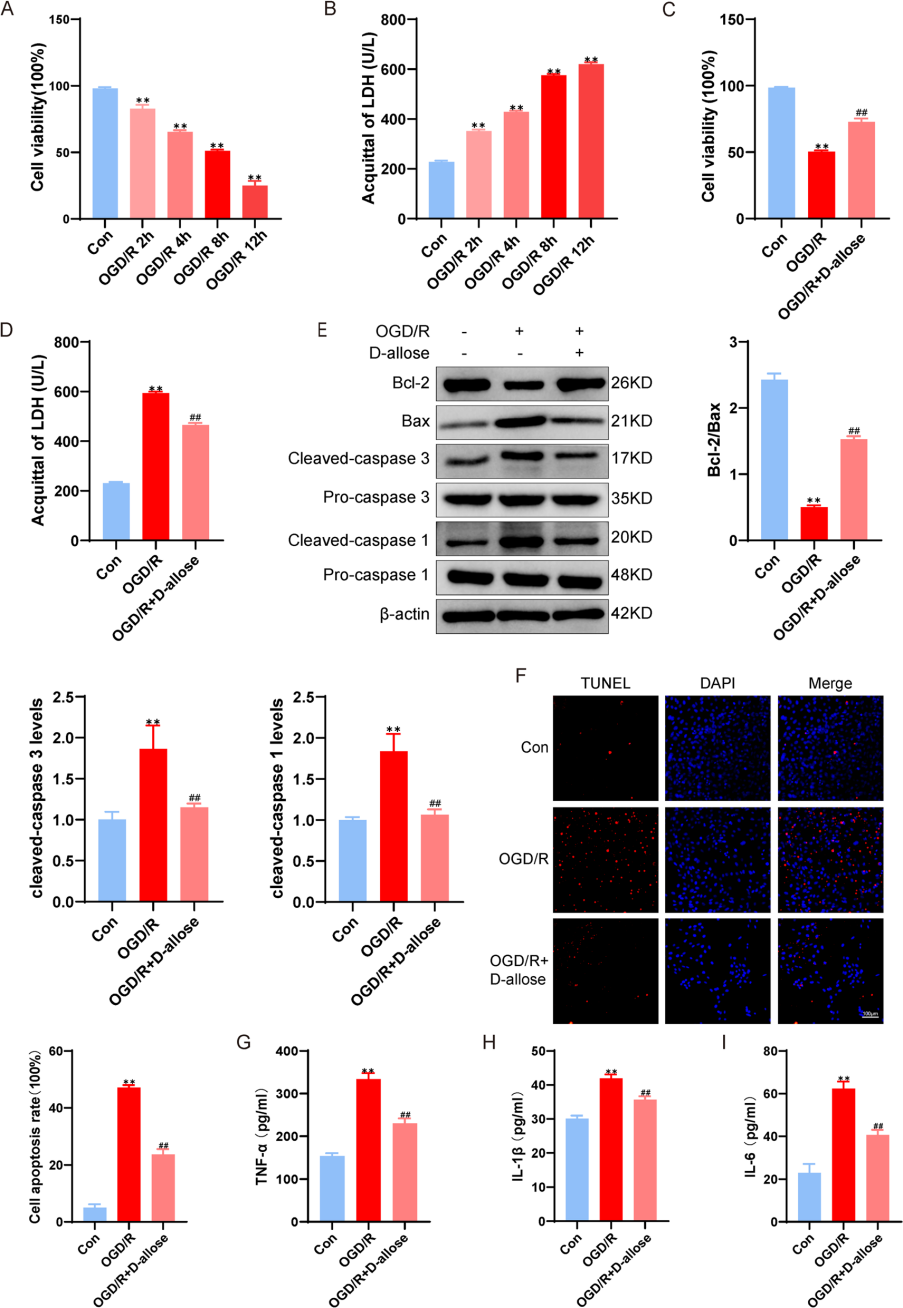
Effects of D-allose on cytotoxicity and apoptosis induced by OGD/R in HT-22 cells. **A** HT-22 cells were subjected to OGD damage for different times, and cell viability was determined by CCK-8 assays. **B** HT-22 cells were subjected to OGD/R injury, and LDH levels were measured. **C** Cell viability was determined by CCK-8 assays after the administration of D-allose. **D** Detection of LDH levels after D-allose treatment. **E** Effects of D-allose on the expression levels of Bax, Bcl-2, Caspase 3, and Caspase 1 in HT-22 cells subjected to OGD/R. **F** Effects of D-allose on HT-22 cell apoptosis induced by OGD/R, as determined by TUNEL staining.  The effects of D-allose on the levels of TNF-α (**G**), IL-1β (**H**) and IL-6 (**I**) were measured by ELISA kits.  **P<0.01 compared with the Con group; ^##^*P*<0.01 compared with the OGD/R group. The Data were analyzed using one-way ANOVA, and Dunnett’s test was used for post hoc analysis. The data were from three independent experiments

In neurons and other nerve cells, OGD/R induced cytotoxicity and caused further. Here, we observed increased LDH release and decreased cell viability at 2 h, 4 h, 8 and 12 h following OGD/R injury in vitro (Fig. 2A-B), and 1.8 mg/mL D-allose markedly reduced the high levels of LDH release induced by OGD/R injury (*P* < 0.01, Fig. 2C-D). Moreover, treatment with D-allose decreased TUNEL-positive cells and cleaved caspase 3 levels in vitro, and the CCK-8 assay showed increases in cell viability and the Bcl-2/Bax ratio in HT-22 cells subjected to OGD/R injury compared with those in the OGD/R group (*P* < 0.01, Fig. 2E-F). Additionally, compared to those in the Sham group, OGD/R induced a significant increase in cleaved Caspase1, IL-1β, IL-6 and TNF-α levels in vitro (*P* < 0.01, Fig. 2G-I), and D-allose markedly decreased these inflammatory factor levels after cell injury. These results indicate that D-allose protects HT-22 cells from OGD/R-induced cytotoxicity, inflammation and apoptosis.

### Increased Expression of Gal-3 During Ischemia/Reperfusion(I/R) Injury-Induced Inflammation and Neuronal Apoptosis in vivo and in vitro

**Fig. 3:**
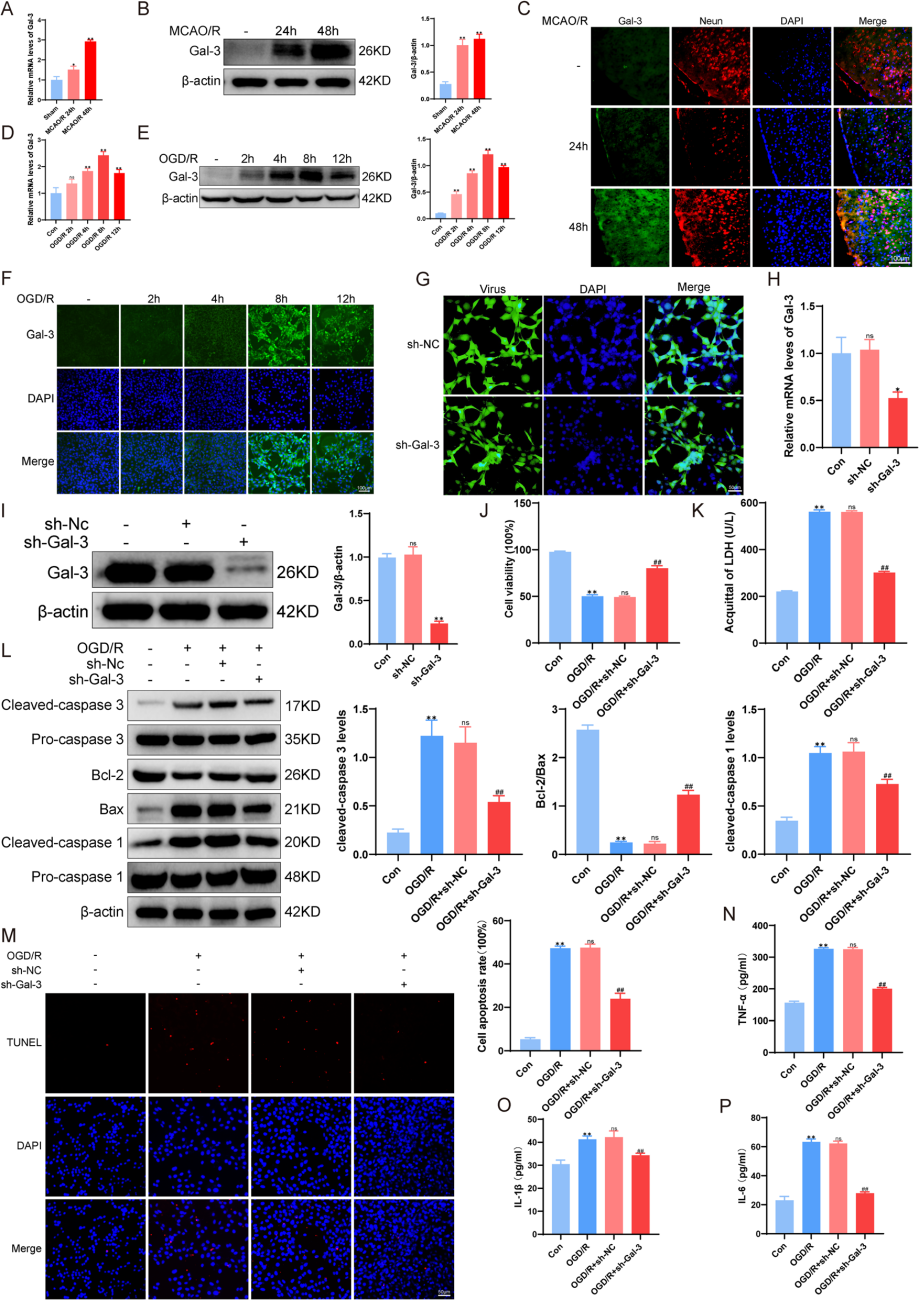
Increased expression of Gal-3 in ischemic/reperfusion(I/R) injury in vivo and in vitro. **A** Changes in Gal-3 mRNA levels in brain tissue 24 and 48 h after MCAO, as measured by RT-PCR. **B** Protein expression of Gal-3 protein content in brain tissue 24 and 48 h after MCAO, as measured by Western blotting. **C** Observation of Gal-3 expression in brain tissue 24 and 48 h after MCAO by immunofluorescence analysis. **D** Detection of Gal-3 mRNA expression in HT-22 cells after different lengths of OGD/R injury using RT-PCR. **E** Examination of Gal-3 expression in HT-22 cells after different durations of OGD/R injury by using Western blotting. **F** Observation of Gal-3 expression in HT-22 cells after different durations of OGD/R injury, as measured by immunofluorescence analysis. **G** IF was used to observe lentiviral transfection. **H** Detection of Gal-3 mRNA expression after lentiviral transfection. **I** Detection of Gal-3 protein expression after transfection with the lentivirus. **J** CCK-8 assay to detect cell viability after transfection with the lentivirus. **K** Detection of LDH levels after lentiviral transfection. **L** Western blot analysis of caspase 3, Bax, Bcl-2, and caspase 1 protein expression after LV transfection. **M** TUNEL staining was used to observe HT-22 cell apoptosis after transfection with different lentiviruses. **N**-**P** The expression levels of inflammatory factors in HT-22 cells transfected with the lentivirus and then damaged by OGD/R were measured using ELISA kits. ** *P*<0.01 compared with the Con group; ^ns^*P*>0.05, ^##^*P*<0.01 compared with the OGD/R group. The data were analyzed using one-way ANOVA, and Dunnett’s test was used for post hoc analysis. The data were from three independent experiments

As an important inflammatory factor, Gal-3 participates in inflammation, oxidative stress, cell apoptosis and pyroptosis in the brain. Compared with those in the Sham group, the mRNA and protein expression levels of Gal-3 increased steadily, peaked at 48 h after MCAO/R (*P* < 0.01, Fig. 3A-B), and well increased at 2 h, 4 h, 8 and 12 h, peaking at 8 h following OGD/R (*P* < 0.01, Fig. 3D-E). Therefore, 48 h after MCAO/R and the 8 h after ODG/R were used in the following experiments. Moreover, similar results were observed by immunohistochemical staining in vitro and in vivo. Compared with that in the Sham group, Gal-3 was located in the cytoplasm and nuclei of neurons and HT-22 cells, and Gal-3 positive-cells were increased 48 h after MCAO/R and 8 h after OGD/R (*P* < 0.01, Fig. 3C, F). These results suggest that Gal-3 is expressed in neurons but not neuroimmune cells and may play a crucial role in inflammation-induced neuronal injury and death.

To further evaluate the role of Gal-3 in OGD/R-induced HT-22 cell injury, lentivirus-based Gal-3-shRNA vectors were used to knock out (KO) the expression of Gal-3 (*P* < 0.01, Fig. 3G). Compared with those in the control group, Gal-3-shRNA significantly decreased Gal-3 mRNA and protein levels by 50% and 70%, respectively in HT-22 cells (*P* < 0.05, Fig. 3H-I). Then, we evaluated the neuroprotective effect of Gal-3 deficiency against OGD/R-induced HT-22 cell injury. As shown in Fig. 3J-P, increases in cell viability and the Bcl-2/Bax ratio and decreases in LDH release, cleaved Caspase 3 levels and TUNEL-positive neurons, as well as levels of cleaved Caspase1, IL-1β, IL-6 and TNF-α, were observed in the OGD/R + sh-Gal-3 group compared to the control group (*P* < 0.01), suggesting that Gal-3 was a key mediator of OGD/R-induced cytotoxicity and apoptosis in HT-22 cells.

### 3.4. Gal-3 Directly Binds with TLR4 in vitro

**Fig. 4:**
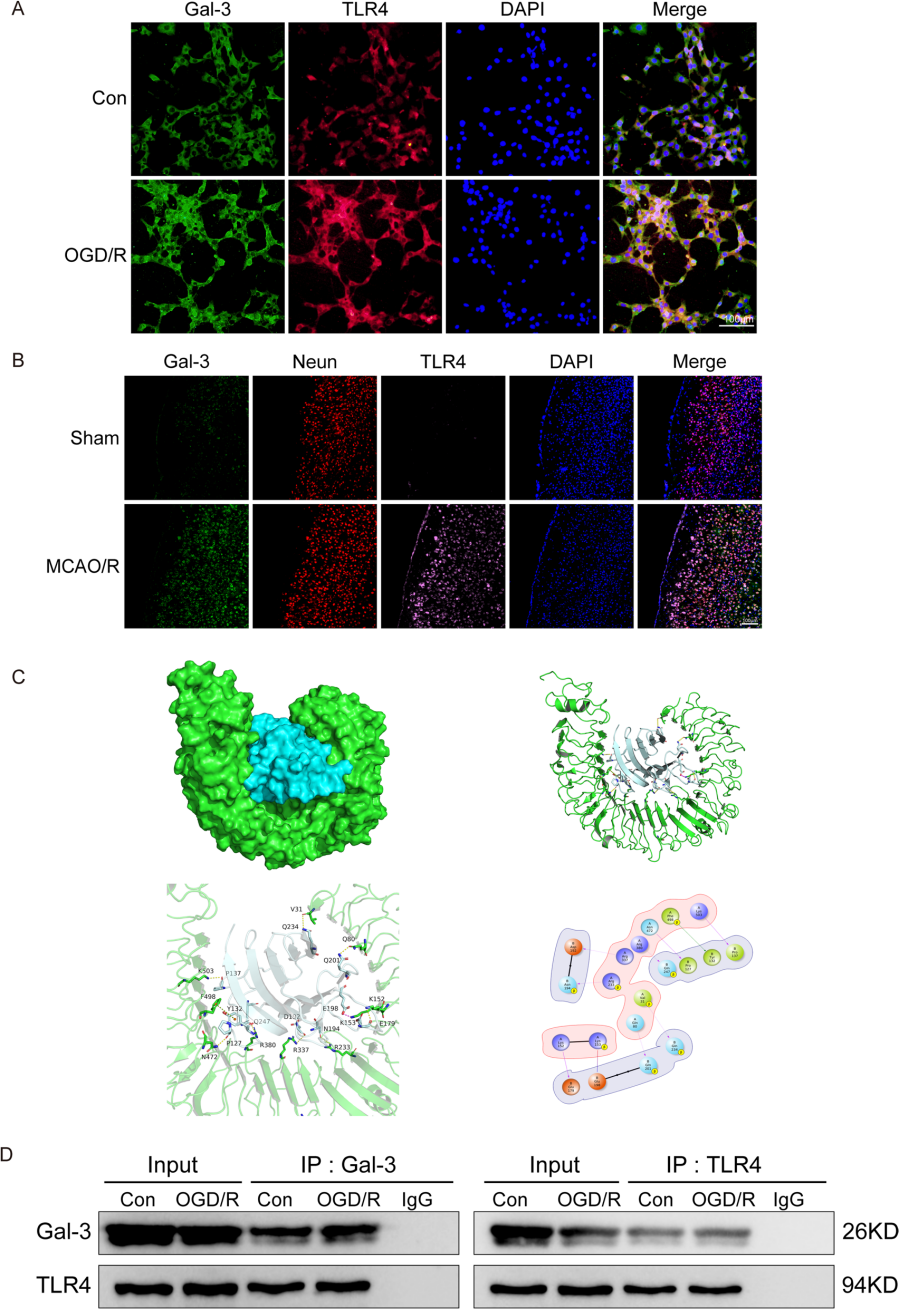
Gal-3 directly binds with TLR4 in vitro.  **A** Immunofluorescence analysis of the colocalization of Gal-3 and TLR4 in HT-22 cells. **B** Immunofluorescence analysis of the colocalization of Gal-3, neurons, and TLR4 in brain tissue. **C** Analysis of protein structure using 3D structure prediction techniques. A mock structural map of the binding of Gal-3 (cyan) to TLR4 (green) was obtained, as well as the specific binding sites.  **D** Immunoblot showing the presence of Gal-3 and TLR4 in an immune complex formed after the pull-down of TLR4 and Gal-3 in HT-22 cells in the con group and OGD group

We examined the relationship between Gal-3 and TLR4 in vitro, and Gal-3 colocalized with and bound to TLR4, as shown by co-IP assays, and the colocalization of Gal-3 and TLR4, which was examined by immunofluorescence staining, was increased after IS (Fig. 4A-B, D). These results suggested that Gal-3 could influence the effects of TLR4 on the inflammatory response and apoptosis in CNS diseases. To clarify the possible structural and functional interaction regions and sites between Gal-3 and TLR4, their binding effects were examined by computer-aided molecular docking, and the two proteins formed a wide range of interactions, mainly including hydrogen bonding, salt bridging and π-π stacking. The salt bridge was formed between the positively charged side chain of the alkaline amino acid Lys153 and the negatively charged carboxylic acid side chain group of the acidic amino acid Glu198. The salt bridge involved the superposition of hydrogen bonds and charges, which belong to a strong ion-type interaction. The π-π stacking effect involved the side chain benzene ring charge center of Phe498 (F498) on TLR4 and the phenol charge center of Tyr132 (Y132) on the Gal-3 protein (Fig. 4C). These data indicate that Gal-3 and TLR4 directly interact based on their physical structures.

### Gal-3 Activates the TLR4/ PI3K/AKT Signaling axis in vitro

**Fig. 5:**
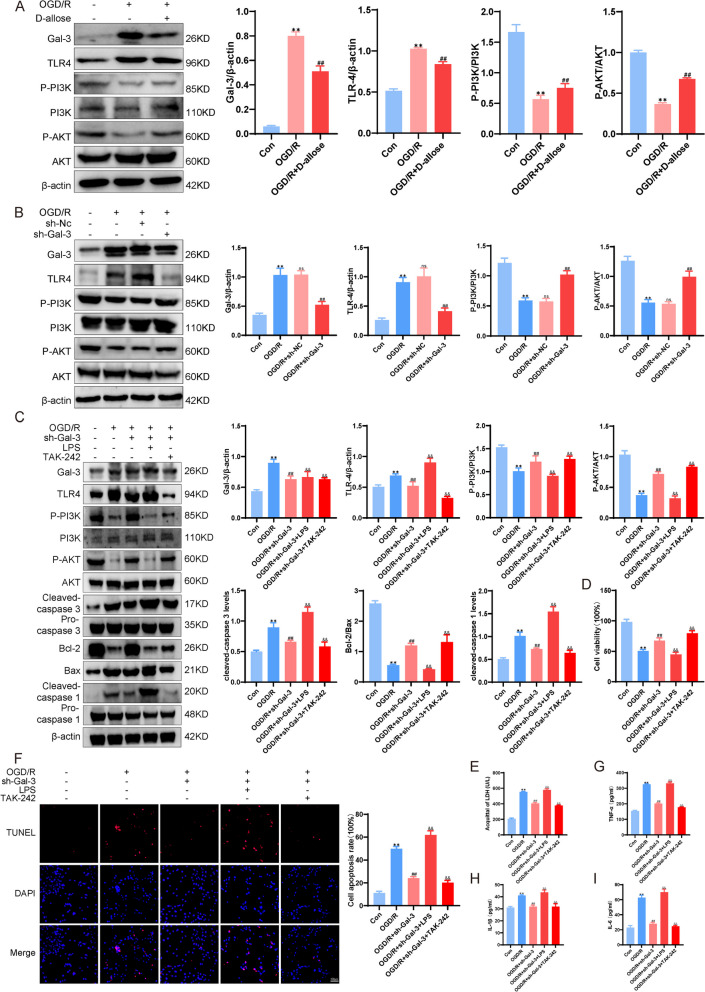
Gal-3 activates the TLR4/ PI3K/AKT signaling axis in vitro. **A** Western blot analysis of the expression of related proteins in the Gal-3-TLR4-PI3K-AKT pathway after the administration of D-allose. **B** Expression of related proteins in the Gal-3-TLR4-PI3K-AKT pathway after lentiviral transfection, as detected by Western blotting. **C** Western blot analysis of the expression of signaling pathways and apoptosis-related proteins after transfection with the Gal-3 lentivirus and the administration of agonists and inhibitors of TLR4.  **D** Detection of HT-22 cell viability by CCK-8 assays after transfection with the Gal-3 lentivirus and the administration of agonists and inhibitors of TLR4. **E** Measurement of LDH levels in HT-22 cells.  **F** TUNEL staining was used to observe apoptosis in each group of HT-22 cells.  **G**-**I** ELISA kits were used to detect the levels of secreted inflammatory factors in the groups of cells. **P<0.01 compared with the Con group; ^ns^*P*>0.05, ^##^*P*<0.01 compared with the OGD/R group; ^&&^*P*<0.01 compared with the OGD/R + sh-Gal-3 group. Data were analyzed using one-way ANOVA and Dunnett’s test was used for post hoc analysis. Data were from three independently conducted experiments

A previous study showed that Gal-3 increased the RNA and protein levels of TLR4 in WD. To further investigate the effect of Gal-3 on the TLR4 signaling pathway and the mechanism, we treated HT-22 cells with a Gal-3-shRNA vector, lipopolysaccharide (LPS) (1 µg/ml), a TLR4 activator and the TLR4 inhibitor TAK-242 (10 µg/ml). We first found that OGD/R injury caused high protein expression of TLR4 and reduced the phosphorylation of PI3K (p-PI3K) and AKT (p-AKT) compared with those in the control group (*P* < 0.01, Fig. 5A). Transfection of HT-22 cells with the Gal-3 shRNA vector significantly decreased TLR4 protein expression and increased the expression levels of p-PI3K and p-AKT after OGD/R compared to those in the OGD/R group (*P* < 0.01, Fig. 5B). In cells transfected with the Gal-3 KO vector, compared with those in the OGD/R group and OGD/R + sh-Gal-3 group, cell viability and the expression of Bcl-2/Bax, p-PI3K and p-AKT (*P* < 0.01, Fig. 5C, D) were reduced, but the release of LDH, the levels of Gal-3, TLR4, cleaved Caspase3, and the ratio of cell apoptosis (*P* < 0.01, Fig. 5C, E) were increased in HT-22 cells treated with LPS. In contrast, HT-22 cells treated with TAK-242 had significantly increased viability and levels of Bcl-2/Bax, p-AKT and p-PI3K (*P* < 0.01, Fig. 5C, D), and decreased LDH release, Gal-3 and TLR4 protein expression, as well as cleaved Caspase 3 and TUNEL-positive cells (*P* < 0.01, Fig. 5C, E, F) compared with those in the OGD/R group or damaged cells transfected with the vector only. Moreover, the opposite change in expression and a significant increase or decrease in the peak values of cleaved Caspase1, IL-1β, IL-6 and TNF-α (*P* < 0.01, Fig. 5C, G-I) were observed in the OGD/R + sh-Gal-3 + LPS group and OGD/R + sh-Gal-3 + TAK-242 group. These results demonstrated that the interaction of Gal-3 with TLR4 and activation of the TLR4/PI3K/AKT signaling axis aggravated OGD/R-induced inflammatory damage and apoptosis in vitro.

### D-allose Regulates the TLR4/ PI3K/AKT Pathway through Gal-3 to Protect HT-22 Cells from OGD/R-induced Injury

**Fig. 6:**
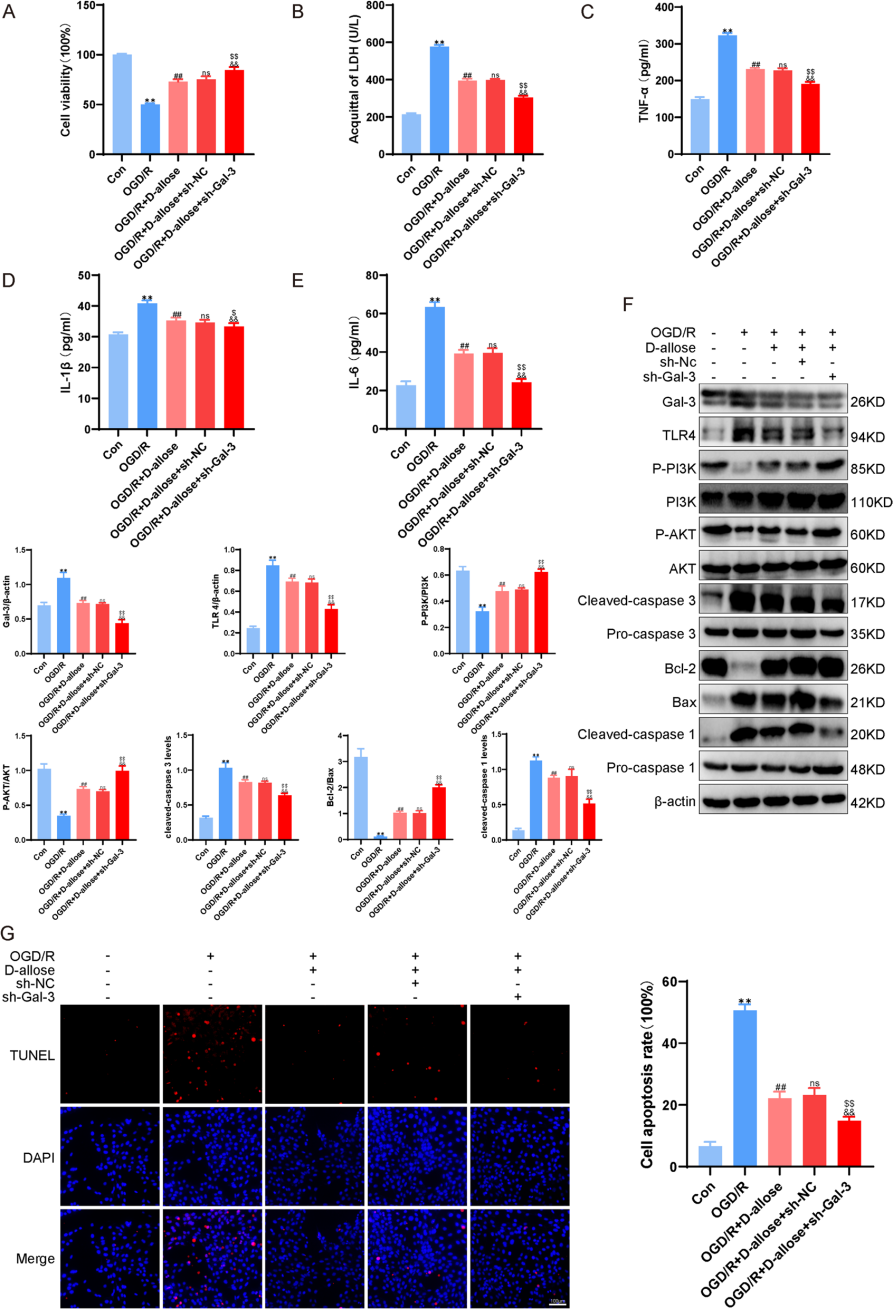
D-allose regulates the TLR4/ PI3K/AKT pathway via Gal-3 to protect HT-22 cells from OGD/R-induced injury. **A** Detection of HT-22 cell viability after transfection with LV and D-allose treatment, as determined by CCK-8 assays. **B** Measurement of LDH levels in HT-22 cells after transfection with the lentivirus and D-allose treatment. **C**, **D**, **E** Analysis of relevant inflammatory factors in each group of HT-22 cells by ELISA. **F** Detection of apoptotic proteins such as Bax and related proteins in the Gal-3-TLR4 signaling pathway by Western blot analysis. **G** TUNEL staining to observe apoptotic cells in each group. **P<0.01 compared with the Con group; ^##^*P*<0.01 compared with the OGD/R group; ^ns^*P*>0.05, ^&&^*P*<0.01 compared with the OGD/R + D-allose group; ^$^*P*<0.05, ^$$^*P*<0.01 compared with the OGD/R + sh-NC group. The data were analyzed using one-way ANOVA and Dunnett’s test was used for post hoc analysis. The data were from three independent experiments

To examine whether Gal-3 and the TLR4/ PI3K/AKT signaling axis are involved in the effect of D-allose on HT-22 cells, cytotoxicity, the inflammatory response and apoptosis were determined by Western blot and immunoblot analysis. Following transfection with sh-Gal-3 or sh-NC, HT-22 cells were treated with D-allose. As shown in Fig. 6A-B, the viability and LDH release of HT-22 cells transfected with sh-Gal-3 were higher than those of HT-22 cells transfected with sh-NC (*P* < 0.01), while the expression of the inflammatory factors IL-1β, IL-6 and TNF-α (*P* < 0.01, Fig. 6C-E) in HT-22 cells transfected with sh-Gal-3 was lower than that in HT-22 cells transfected with sh-NC. Moreover, compared with the OGD/R + D-allose + sh-NC group, and the OGD/R + D-allose + sh-Gal-3 group exhibited higher levels of p-PI3K, p-AKT and Bcl-2/Bax and lower levels of Gal-3, TLR4, cleaved Caspase3, cleaved Caspase1 and TUNEL-positive cells (*P* < 0.01, Fig. 6F-G). Taken together, these results indicated that D-allose could inhibit TLR4/PI3K/AKT-induced cytotoxicity, inflammatory damage and apoptotic cell death by inducing Gal-3.

### D-allose Inhibits TLR4/PI3K/AKT Signaling to Attenuate Neuroinflammation and Apoptosis by Inhibiting Gal-3 after MCAO/R Injury

**Fig. 7:**
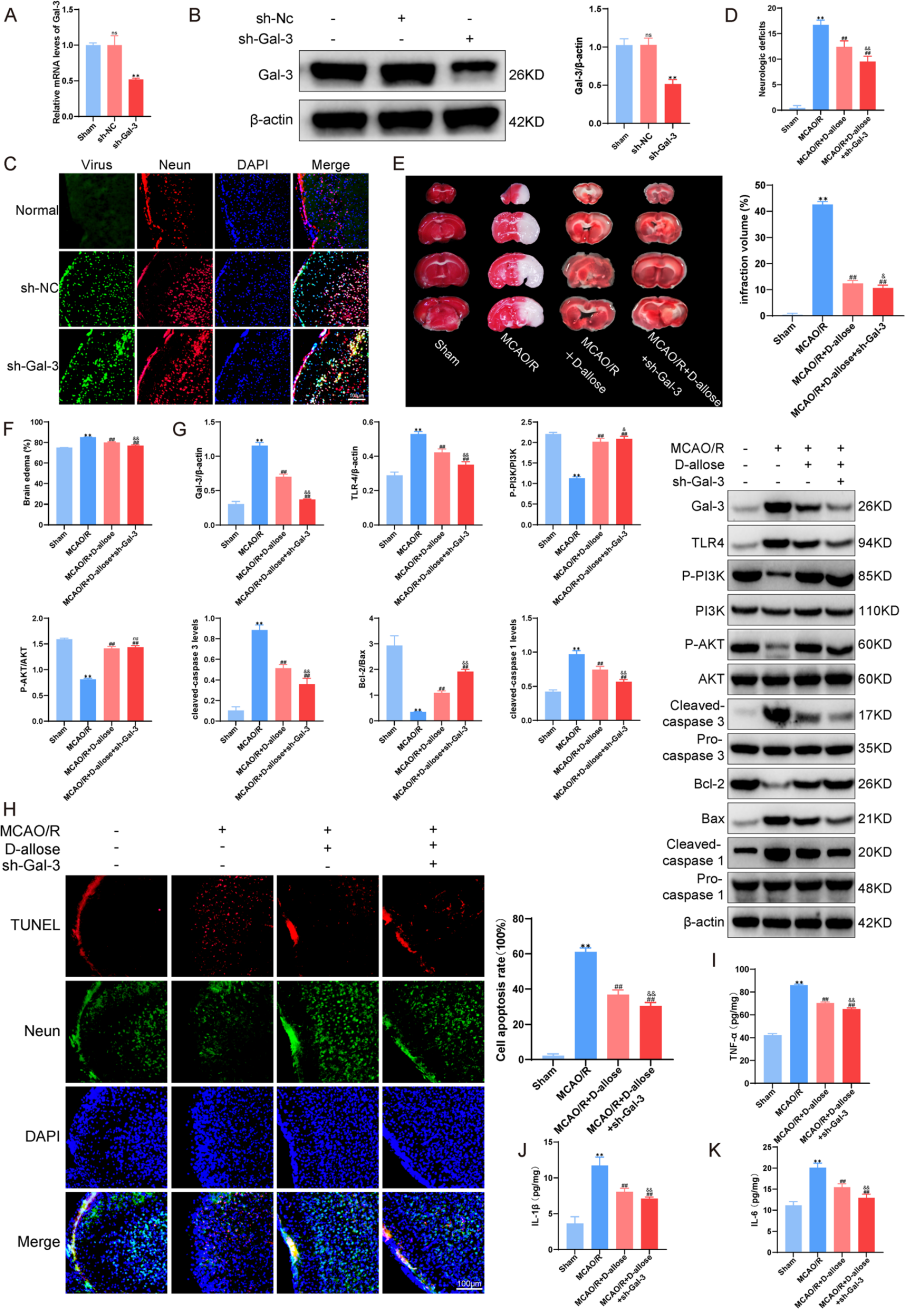
D-allose inhibits TLR4/PI3K/AKT signaling to attenuate neuroinflammation and apoptosis by inhibiting Gal-3 after MCAO/R injury. **A** RT-PCR analysis of the mRNA expression of Gal-3 in mice after transfection with AAV.  **B** Western blotting was performed to detect the protein expression of Gal-3 in mice after AAV transfection. **C** Immunofluorescence analysis of mice transfected with AAV. **D** The transfected mice were subjected to MCAO injury and administered D-allose treatment, and their neurological function was scored. **E** TTC staining was used to observe the changes in infarct volume. **F** Brain edema was observed in each group of mice after injury. **G** Western blotting was performed to detect the expression of Gal-3-TLR4 signaling pathway and apoptosis proteins in each group of mice.  (H) TUNEL staining to observe neuronal apoptosis in mouse brain tissue. **I**, **J**, **K** ELISA kits were used to detect the secretion of TNF-α, IL-1β and IL-6 in brain tissue.  ^ns^*P*>0.05, **P<0.01 compared with the Sham group; ^##^*P*<0.01 compared with the MCAO group; ^ns^*P*>0.05, ^&^*P*<0.05, ^&&^*P*<0.01 compared with the MCAO + D-allose group. Data were analyzed using one-way ANOVA, and Dunnett’s test was used for post hoc analysis. The data were from three independent experiments

Based on our novel findings of the neuroprotective effect of D-allose associated with Gal-3 and the TLR4/PI3K/AKT signaling pathway in vitro, we stereotactically injected AAV-based shRNA Gal-3 into the cortical areas of mice before MCAO/R. First, compared with those in the MCAO/R + sh-Nc group, the mRNA and protein levels of Gal-3 were decreased in Gal-3 KO mice after MCAO/R injury (*P* < 0.01, Fig. 7A-C). The results showed that the downregulation of Gal-3 attenuated the infraction volume ratio and brain edema and enhanced the protective effects of D-allose. Similar to the change in the infraction volume ratio, the same effects on neurological deficits were observed in the different groups (*P* < 0.01, Fig. 7D-F). Moreover, after treatment with D-allose, Gal-3 KO mice had significantly increased phosphorylation of PI3K and AKT and reduced expression of Gal-3 and TLR4 compared to wild-type mice following MCAO/R injury (*P* < 0.01, Fig. 7G). These mice also exhibited decreased levels of cleaved Caspase3, cleaved Caspase1 (Fig. 7G), and TUNEL-positive cells, IL-1β, IL-6 and TNF-α (Fig. 7H-K). Combined with these in vitro data, these results suggest that D-allose inhibits TLR4/PI3K/AKT signaling to attenuate neuroinflammation and apoptosis and ameliorate neurological deficits by inhibiting Gal-3 in the context of secondary IS injury.

**Fig. 8:**
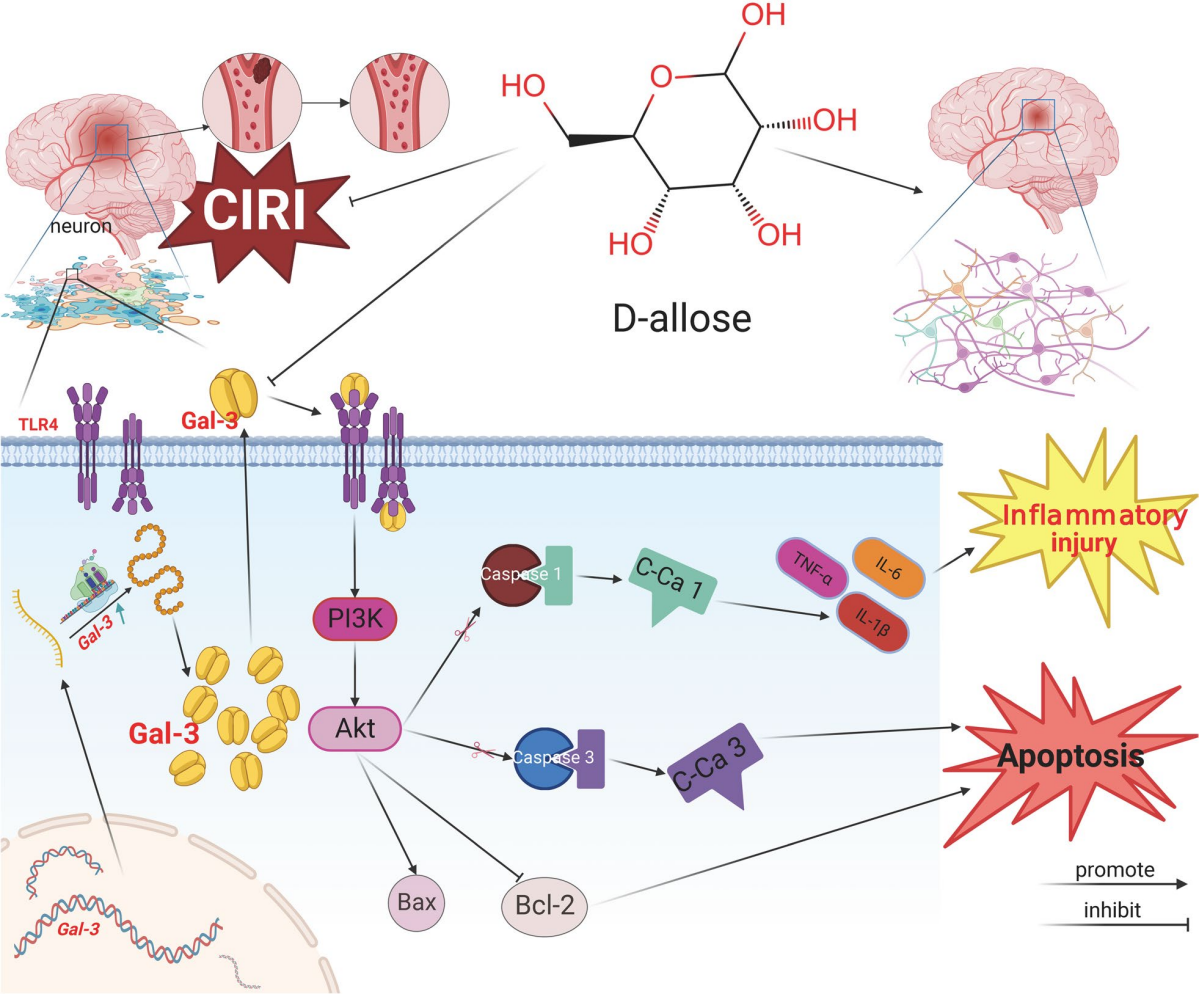
Potential mechanisms by which D-allose exerts neuroprotective effects against I/R-induced brain injury in mice and HT-22 cells. D-allose protects the brain from ischemia reperfusion-induced apoptosis and inflammation by suppressing Gal-3 expression and transcriptional processes that inhibit TLR4 and activate PI3K/AKT phosphorylation

## Discussion

IS is characterized by the interruption or sudden restriction of cerebral blood flow in the cortex, hippocampus and basal ganglia of the brain, but the complex set of events, taking place in the ischemic penumbra, which is an area of hypoxia and glucose deprivation, after the onset of IS, is called the ischemic cascade or secondary brain injury (SBI) and is the main cause of high disability and mortality. In recent decades, evidence has highlighted that the loss of ionic homeostasis, energy failure, excessive excitatory amino acid excitotoxicity, increased oxidative stress and immune responses stimulate neuronal damage and death in the ischemic penumbra [47, 48] in inflammation in neuroimmune cells and the induction of neuronal apoptosis are two main pathophysiological mechanisms of SBI after IS [49]. Previous studies have shown that many drugs extracted from plants that target inflammatory injury and apoptotic cell death are useful treatments for IS-induced SBI [50]. D-allose, which is a safe “rare sugar” for consumption by mammals, is a C-3 epimer of glucose that is an ultralow calorie sugar with no toxicity that exhibits unique and broad-spectrum health benefits and physiological functions in food systems, clinical treatment, and health care, including antitumor [51], antiosteoporotic [52], anti-inflammatory [53], cryoprotective [54], antihypertensive [55], neuroprotective [16], and immunosuppressive [56] functions, as well as antioxidative properties by modulating the generation of reactive oxygen species (ROS). D-allose was developed as a potential drug for treating cancer and ischemia/reperfusion (I/R) injury. However, the relationships between D-allose, inflammation and apoptosis have not been elucidated. Overall, the current study showed several novel findings. First, D-allose administration after IS was beneficial for reducing cytotoxicity, inflammation and neuronal apoptosis and improving neurological function in vitro and in vivo. Second, Gal-3 could directly bind with TLR4 and activate the TLR4/PI3K/AKT signaling pathway in OGD/R and MCAO/R models. Third, D-allose regulated TLR4/PI3K/AKT signaling to attenuate neuroinflammation and apoptosis by inhibiting Gal-3 after IS in HT-22 cells and mice.

IS often causes inflammatory injury, neuronal death and neurological deficiency. Accumulating evidence indicates that D-allose exerts many distinct cytoprotective effects on I/R injuries in different organs. However, the mechanism underlying the protective effect of D-allose on I/R injury in vitro and in vivo are still largely unclear. Gao et al. reported that D-allose reduced the infarct volume in rat brain following acute cerebral I/R injury [16]. In this study, treatment with D-allose (0.4 mg/g, intraperitoneal) reduced brain insults (infarction, including ischemic penumbra and atrophy volume) and behavioral deficits after focal cerebral ischemia, suggesting that D-allose protects the brain from I/R damage in mice. Previous studies have indicated that D-allose reduces the production of ROS in many diseases [11, 16, 37, 57]. In our previous study, we concluded that D-allose protected brain microvascular endothelial cells from hypoxic/reoxygenated injury by inhibiting the UPR pathway and attenuating eIF2αphosphorylation and endoplasmic reticulum stress [58]. Moreover, previous reports showed the anti-inflammatory effects of D-allose and inhibition of LPS-induced increases in TNF-α, tissue cytokine-induced neutrophil chemoattractant-1 and myeloperoxidase concentrations, and subsequent neutrophil-mediated renal, cerebral and skin injury. Interestingly, there are contrary results on the effect of D-allose on programmed apoptosis in a variety of cells. On the one hand, D-allose induces tumor cell apoptosis in lung cancer [59, 60]. On the other hand, D-allose decreases apoptotic cell death in I/R-induced injury in different organs and tissues. Moreover, some studies have demonstrated that D-allose exerts its anti-inflammatory and antiapoptotic effects to protect the liver [61], kidney [13], retina [62] and skin flaps [63] from I/R injury. Our previous studies provided evidence that D-allose may exert therapeutic effects against cerebral I/R injury by attenuating BBB disruption, inflammatory damage and apoptosis via PPARγ-dependent regulation of NF-κB [37].

Consistently, in the present study, we found that intraperitoneal administration of D-allose markedly reduced neuronal cytotoxicity, as indicated by the release of LDH and the viability of HT-22 cells, decreased the infarct volume ratio and brain edema, and improved neurological dysfunction in mice after I/R injury. Additionally, we found that D-allose treatment significantly reduced cerebral I/R-induced neuronal apoptosis by inhibiting the inflammatory response. Taken together, our findings suggested that D-allose protected against apoptotic cell death by mediating anti-inflammatory effects after I/R injury. Compared to only commercially available medicines used for IS, such as tissue plasminogen activator (tPA), D-allose exerts more efficient neuroprotective effects on SBI in mice and can reduce brain insults and behavioral deficits, decrease inflammatory injury, promote recover from injury and alleviate apoptosis and pyroptosis. More importantly, D-allose, which has the same sweet taste and bulk properties as sugar, is not metabolized in the human body, and therefore has no known health effects on human. Thus, D-allose can be added to food to prevent and treat IS.

The mechanism of the protective effect of D-allose in vitro and in vivo after IS was examined. Gal-3 belongs to the Type three β-galectin binding lectin family and plays an important exacerbating role in autoimmune, inflammatory, and malignant diseases and I/R injury. D-allose up, -or downregulated apoptosis in different diseases, and the function of Gal-3 in apoptotic cell death is also controversial. Some reports have shown that Gal-3 regulates ischemic stroke by inhibiting neuronal apoptosis in rats, but other studies have indicated that Gal-3 can modulate glioma by enhancing tumor cell apoptosis, and increased levels of Gal-3 are found in some CNS diseases [26, 64–66]. Furthermore, whether Gal-3 is a proinflammatory or anti-inflammatory factor remains unclear. In this study, compared with that in the I/R group, D-allose treatment significantly decreased the expression level of Gal-3, the apoptotic markers, Bax and cleaved-Caspase3, and inflammatory mediators, such as cleaved Caspase1, IL-1β, IL-6 and TNF-α, in the brain and HT-22 cells, suggesting that the reduced expression of Gal-3 induced by D-allose could mediate the neuroprotective effects we observed in this study. To confirm this, a Gal-3 knockdown experiment was performed. The results showed that in Gal-3 knockdown brains and HT-22 cells, the anti-inflammatory and antiapoptotic effects of D-allose were enhanced. These results suggested that the increase in Gal-3 expression induced inflammatory insults and substantial neuronal apoptosis in vitro and in vivo after I/R injury, and the protective effects of D-allose on IS might occur via the Gal-3 pathway.

Furthermore, the TLR family member TLR4 mediates several cellular processes, such as apoptosis, oxidative stress, infection-induced inflammation and sterile inflammation via endogenous molecules [29, 67]. Recent evidence has shown that as a ligand of TLR4, Gal-3 activates TLR4/NF-κB signaling to promote cell proliferation and migration in lung adenocarcinoma reduce myocardial injury and improve cardiac dysfunction by inhibiting apoptosis [68]. Other studies revealed the TLR4-mediated proinflammatory and apoptotic effects of Gal-3 [22]. Additionally, PI3K/AKT signaling is regulated by ROS production and the products of metabolism and participates in the regulation of oxidative stress, the inflammatory response, and apoptosis in I/R injury [69]. TLR4 is an upstream regulator of the PI3K/AKT axis and upregulates p-PI3K and p-AKT levels, resulting in the activation of TLR4/PI3K/AKT signaling, which promotes proliferation and migration during the development of cardiac hypertrophy [70]. Recent research has revealed that Gal-3 is involved in the modulation of angiogenesis and apoptosis by inhibiting AKT signaling to enhance neurovascular protection and functional recovery after IS [26]. In this study, we first showed that Gal-3 could directly bind to TLR4, and the loss of Gal-3 reduced the rate of apoptosis and the levels of the apoptotic proteins, Bax, cleaved Caspase 3 and cleaved Caspase 1 and the proinflammatory cytokines, IL-1β, IL-6 and TNF-α, which inhibited the TLR4/PI3K/AKT signaling pathway in the OGD/R and MCAO/R models. Therefore, compared with I/R injury + Gal-3 KO in vitro and in vivo, the anti-inflammatory and anti-apoptotic effects of D-allose were suppressed in Gal-3 KO HT-22 cells and mouse cerebral tissue treated with the TLR4 agonist LPS. To the best of our knowledge, this is the first report to confirm that D-allose treatment inhibits the TLR4/PI3K/AKT signaling pathway to significantly reduce cerebral I/R-induced neuroinflammation and neuronal apoptosis by attenuating Gal-3 in vitro and in vivo.

There are some limitations in our study. Although HT-22 cells have been widely used as an in vitro IS model of neuronal function, different results about the effects of D-allose on IS can be observed in primary neurons. The exact mechanism by which Gal-3 activates TLR4/PI3K/AKT signaling was not identified. Therefore, we need to perform more studies on other neuronal cells to mimic the IS model in vitro to identify D-allose-mediated neuroprotection in I/R injury and shed light on the anti-inflammatory and antiapoptotic mechanism. Moreover, in this study, we chose a MCAO/R model to mimic human I/R injury in C57BL/6 N mice, but rats and monkeys can be used to produce MCAO/R models, and more realistic results can be obtained. Additionally, there are many types of cell death, including necrosis, programmed necrosis, apoptosis, pyroptosis, ferroptosis, and copper-induced cell death, after IS. We only studied apoptosis in the present study, and we will identify the relationship between D-allose and other types of cell death in subsequent studies. Moreover, there were some sources of bias in our study; for example, by using intraperitoneal injection, the plasma concentration of D-allose is unclear, and the Gal-3 knockdown AAV was injected the cerebrum, which cannot guarantee the knockout efficiency of Gal-3 in all brain cells. Therefore, it is best to monitor the plasma concentration of D-allose and use Gal-3 conditional knockout animals to eliminate some bias.

In conclusion, our findings in mouse brains and HT-22 cells suggest that D-allose attenuates I/R-induced brain damage, neuronal cytotoxicity and apoptosis by reducing inflammation. This protective effect may be largely due to the Gal-3/TLR4 complex inhibiting the TLR4/PI3K/AKT signaling axis in SBI during IS (Fig. 8 Created with BioRender.com.). With increased understanding of the mechanisms of cerebral SBI associated with I/R damage and the functions of D-allose, which is a monosaccharide that has few side effects, no metabolism in the body, and significant anti-inflammatory, antiapoptotic and antipyroptotic effects after IS, D-allose may serve as a promising candidate for the prevention and treatment of IS in patients.

## Data Availability

The datasets used and/or analyzed during the current study are available from the corresponding author on reasonable request.

## References

[1] Hankey GJ (2017). Stroke. Lancet.

[2] Wang YJ, Li ZX, Gu HQ, Zhai Y, Zhou  Q, Jiang Y, Zhao XQ, Wang YL, Yang X, Wang CJ (2019). China National Clinical Research Center for Neurological. China Stroke Statistics: an update on the report from the National Center for Healthcare Quality Management in Neurological Diseases, Diseases, the Chinese Stroke Association, National Center for Chronic and Non-communicable Disease Control and Prevention, Chinese Center for Disease Control and Prevention and Institute for Global Neuroscience and Stroke Collaborations. Stroke Vasc Neurol.

[3] Nour M, Scalzo F, Liebeskind DS (2013). Ischemia-reperfusion injury in Stroke. Interv Neurol.

[4] DeLong JH, Ohashi SN, O’Connor KC, Sansing LH (2022). Inflammatory responses after ischemic Stroke. Semin Immunopathol.

[5] Zhang G, Li Q, Tao W, Qin P, Chen J, Yang H, Chen J, Liu H, Dai Q, Zhen X (2023). Sigma-1 receptor-regulated efferocytosis by infiltrating circulating macrophages/microglial cells protects against neuronal impairments and promotes functional recovery in cerebral ischemic Stroke. Theranostics.

[6] Xu Q, Zhao B, Ye Y, Li Y, Zhang Y, Xiong X, Gu L (2021). Relevant mediators involved in and therapies targeting the inflammatory response induced by activation of the NLRP3 inflammasome in ischemic Stroke. J Neuroinflammation.

[7] Puig B, Brenna S, Magnus T. Molecular Communication of a Dying Neuron in Stroke. Int J Mol Sci. 2018;19(9):2834. 10.3390/ijms19092834 PMC6164443 30235837

[8] Tsivgoulis G, Katsanos AH, Sandset EC, Turc G, Nguyen TN, Bivard A, Fischer U, Khatri P (2023). Thrombolysis for acute ischaemic Stroke: current status and future perspectives. Lancet Neurol.

[9] Szabó Í, Varga VÉ, Dvorácskó S, Farkas AE, Körmöczi T, Berkecz R, Kecskés S, Menyhárt Á, Frank R, Hantosi D et al. N,N-Dimethyltryptamine attenuates spreading depolarization and restrains neurodegeneration by sigma-1 receptor activation in the ischemic rat brain. Neuropharmacology. 2021;192:108612. 10.1016/j.neuropharm.2021.108612 34023338

[10] Chen Z, Chen J, Zhang W, Zhang T, Guang C, Mu W (2018). Recent research on the physiological functions, applications, and biotechnological production of D-allose. Appl Microbiol Biotechnol.

[11] Murata A, Sekiya K, Watanabe Y, Yamaguchi F, Hatano N, Izumori K, Tokuda M (2003). A novel inhibitory effect of D-allose on production of reactive oxygen species from neutrophils. J Biosci Bioeng.

[12] Mitani T, Hoshikawa H, Mori T, Hosokawa T, Tsukamoto I, Yamaguchi F, Kamitori K, Tokuda M, Mori N (2009). Growth inhibition of head and neck carcinomas by D-allose. Head Neck.

[13] Ueki M, Taie S, Chujo K, Asaga T, Iwanaga Y, Maekawa N (2007). Inhibitory effect of d-allose on neutrophil activation after rat renal ischemia/reperfusion. J Biosci Bioeng.

[14] Hossain MA, Wakabayashi H, Izuishi K, Okano K, Yachida S, Tokuda M, Izumori K, Maeta H (2006). Improved microcirculatory effect of D-allose on hepatic ischemia reperfusion following partial hepatectomy in cirrhotic rat liver. J Biosci Bioeng.

[15] Hirooka K, Miyamoto O, Jinming P, Du Y, Itano T, Baba T, Tokuda M, Shiraga F (2006). Neuroprotective effects of D-allose against retinal ischemia-reperfusion injury. Invest Ophthalmol Vis Sci.

[16] Gao D, Kawai N, Nakamura T, Lu F, Fei Z, Tamiya T (2013). Anti-inflammatory effect of D-allose in cerebral ischemia/reperfusion injury in rats. Neurol Med Chir (Tokyo).

[17] Jeon SB, Yoon HJ, Chang CY, Koh HS, Jeon SH, Park EJ (2010). Galectin-3 exerts cytokine-like regulatory actions through the JAK-STAT pathway. J Immunol.

[18] Nishikawa H, Suzuki H. Possible role of inflammation and Galectin-3 in Brain Injury after Subarachnoid Hemorrhage. Brain Sci. 2018;8(2):30. 10.3390/brainsci8020030 PMC5836049 29414883

[19] Sciacchitano S, Lavra L, Morgante A, Ulivieri A, Magi F, De Francesco GP, Bellotti C, Salehi LB, Ricci A. Galectin-3: one molecule for an alphabet of Diseases, from a to Z. Int J Mol Sci. 2018;19(2):379. 10.3390/ijms19020379 PMC5855601 29373564

[20] Ekingen E, Yilmaz M, Yildiz M, Atescelik M, Goktekin MC, Gurger M, Alatas OD, Basturk M, Ilhan N (2017). Utilization of glial fibrillary acidic protein and galectin-3 in the diagnosis of cerebral infarction patients with normal cranial tomography. Niger J Clin Pract.

[21] Shin T (2013). The pleiotropic effects of galectin-3 in neuroinflammation: a review. Acta Histochem.

[22] Soares LC, Al-Dalahmah O, Hillis J, Young CC, Asbed I, Sakaguchi M, O’Neill E, Szele FG. Novel Galectin-3 roles in neurogenesis, inflammation and neurological Diseases. Cells. 2021;10(11):3047. 10.3390/cells10113047 PMC8618878 34831271

[23] Jiang HR, Al Rasebi Z, Mensah-Brown E, Shahin A, Xu D, Goodyear CS, Fukada SY, Liu FT, Liew FY, Lukic ML (2009). Galectin-3 deficiency reduces the severity of experimental autoimmune encephalomyelitis. J Immunol.

[24] Mietto BS, Jurgensen S, Alves L, Pecli C, Narciso MS, Assuncao-Miranda I, Villa-Verde DM, de Souza Lima FR, de Menezes JR, Benjamim CF (2013). Lack of galectin-3 speeds wallerian degeneration by altering TLR and pro-inflammatory cytokine expressions in injured sciatic nerve. Eur J Neurosci.

[25] Wesley UV, Vemuganti R, Ayvaci ER, Dempsey RJ (2013). Galectin-3 enhances angiogenic and migratory potential of microglial cells via modulation of integrin linked kinase signaling. Brain Res.

[26] Wesley UV, Sutton IC, Cunningham K, Jaeger JW, Phan AQ, Hatcher JF, Dempsey RJ (2021). Galectin-3 protects against ischemic Stroke by promoting neuro-angiogenesis via apoptosis inhibition and Akt/Caspase regulation. J Cereb Blood Flow Metab.

[27] Fukumori T, Takenaka Y, Yoshii T, Kim HR, Hogan V, Inohara H, Kagawa S, Raz A (2003). CD29 and CD7 mediate galectin-3-induced type II T-cell apoptosis. Cancer Res.

[28] Jayaraj RL, Azimullah S, Beiram R, Jalal FY, Rosenberg GA. Neuroinflammation: friend and foe for ischemic Stroke. J Neuroinflamm. 2019;16(1):142. 10.1186/s12974-019-1516-2 PMC6617684 31291966

[29] Kawai T, Akira S (2007). TLR signaling. Semin Immunol.

[30] Dong X, Wang L, Song G, Cai X, Wang W, Chen J, Wang G (2021). Physcion protects rats against cerebral ischemia-reperfusion Injury via Inhibition of TLR4/NF-kB signaling pathway. Drug Des Devel Ther.

[31] Xu S, Wang J, Jiang J, Song J, Zhu W, Zhang F, Shao M, Xu H, Ma X, Lyu F (2020). TLR4 promotes microglial pyroptosis via lncRNA-F630028O10Rik by activating PI3K/AKT pathway after spinal cord injury. Cell Death Dis.

[32] Huang CY, Deng JS, Huang WC, Jiang WP, Huang GJ. Attenuation of Lipopolysaccharide-Induced Acute Lung Injury by Hispolon in Mice, Through Regulating the TLR4/PI3K/Akt/mTOR and Keap1/Nrf2/HO-1 Pathways, and Suppressing Oxidative Stress-Mediated ER Stress-Induced Apoptosis and Autophagy. Nutrients. 2020;12(6):1742. 10.3390/nu12061742 PMC7352175 32532087

[33] Chen J, Wang Z, Zheng Z, Chen Y, Khor S, Shi K, He Z, Wang Q, Zhao Y, Zhang H (2017). Neuron and microglia/macrophage-derived FGF10 activate neuronal FGFR2/PI3K/Akt signaling and inhibit microglia/macrophages TLR4/NF-kappaB-dependent neuroinflammation to improve functional recovery after spinal cord injury. Cell Death Dis.

[34] Zhang L, Wei Q, Liu X, Zhang T, Wang S, Zhou L, Zou L, Fan F, Chi H, Sun J, Wang D (2021). Exosomal microRNA-98-5p from hypoxic bone marrow mesenchymal stem cells inhibits myocardial ischemia-reperfusion injury by reducing TLR4 and activating the PI3K/Akt signaling pathway. Int Immunopharmacol.

[35] Liu Y, Zhao C, Meng J, Li N, Xu Z, Liu X, Hou S (2022). Galectin-3 regulates microglial activation and promotes inflammation through TLR4/MyD88/NF-kB in experimental autoimmune uveitis. Clin Immunol.

[36] Longa EZ, Weinstein PR, Carlson S, Cummins R (1989). Reversible middle cerebral artery occlusion without craniectomy in rats. Stroke.

[37] Huang T, Gao D, Hei Y, Zhang X, Chen X, Fei Z (2016). D-allose protects the blood brain barrier through PPARgamma-mediated anti-inflammatory pathway in the mice model of ischemia reperfusion injury. Brain Res.

[38] Chen J, Li Y, Wang L, Lu M, Zhang X, Chopp M (2001). Therapeutic benefit of intracerebral transplantation of bone marrow stromal cells after cerebral ischemia in rats. J Neurol Sci.

[39] Chen J, Sanberg PR, Li Y, Wang L, Lu M, Willing AE, Sanchez-Ramos J, Chopp M (2001). Intravenous administration of human umbilical cord blood reduces behavioral deficits after Stroke in rats. Stroke.

[40] Bederson JB, Pitts LH, Germano SM, Nishimura MC, Davis RL, Bartkowski HM (1986). Evaluation of 2,3,5-triphenyltetrazolium chloride as a stain for detection and quantification of experimental cerebral infarction in rats. Stroke.

[41] Hatashita S, Hoff JT, Salamat SM (1988). Ischemic brain edema and the osmotic gradient between blood and brain. J Cereb Blood Flow Metab.

[42] Loo DT (2011). In situ detection of apoptosis by the TUNEL assay: an overview of techniques. Methods Mol Biol.

[43] Kim H, Yoon SC, Lee TY, Jeong D (2009). Discriminative cytotoxicity assessment based on various cellular damages. Toxicol Lett.

[44] Anne Waller H, Kay Savage A (2001). mRNA detection by in situ rt-PCR. Methods Mol Med.

[45] Lin JS, Lai EM (2017). Protein-protein Interactions: Co-immunoprecipitation. Methods Mol Biol.

[46] Keiser MS, Chen YH, Davidson BL (2018). Techniques for Intracranial Stereotaxic injections of Adeno-Associated viral vectors in adult mice. Curr Protoc Mouse Biol.

[47] Guo Q, Kawahata I, Cheng A, Wang H, Jia W, Yoshino H, Fukunaga K. Fatty acid-binding proteins 3 and 5 are involved in the initiation of mitochondrial damage in ischemic neurons. Redox Biol. 2023;59:102547. 10.1016/j.redox.2022.102547 PMC9727700 36481733

[48] Sun S, Lv W, Li S, Zhang Q, He W, Min Z, Teng C, Chen Y, Liu L, Yin J (2023). Smart Liposomal Nanocarrier enhanced the treatment of ischemic Stroke through Neutrophil Extracellular traps and Cyclic Guanosine Monophosphate-Adenosine Monophosphate synthase-stimulator of Interferon genes (cGAS-STING) pathway inhibition of ischemic Penumbra. ACS Nano.

[49] Wang Z, Zhou F, Dou Y, Tian X, Liu C, Li H, Shen H, Chen G (2017). Melatonin alleviates Intracerebral Hemorrhage-Induced secondary brain Injury in rats via suppressing apoptosis, inflammation, oxidative stress, DNA damage, and Mitochondria Injury. Transl Stroke Res.

[50] Wu PF, Zhang Z, Wang F, Chen JG (2010). Natural compounds from traditional medicinal herbs in the treatment of cerebral ischemia/reperfusion injury. Acta Pharmacol Sin.

[51] Noguchi C, Kamitori K, Hossain A, Hoshikawa H, Katagi A, Dong Y, Sui L, Tokuda M, Yamaguchi F (2016). D-Allose inhibits Cancer Cell Growth by reducing GLUT1 expression. Tohoku J Exp Med.

[52] Yamada K, Noguchi C, Kamitori K, Dong Y, Hirata Y, Hossain MA, Tsukamoto I, Tokuda M, Yamaguchi F (2012). Rare sugar d-allose strongly induces thioredoxin-interacting protein and inhibits osteoclast differentiation in Raw264 cells. Nutr Res.

[53] Shinohara N, Nakamura T, Abe Y, Hifumi T, Kawakita K, Shinomiya A, Tamiya T, Tokuda M, Keep RF, Yamamoto T, Kuroda Y (2016). d-Allose attenuates overexpression of inflammatory cytokines after Cerebral Ischemia/Reperfusion Injury in Gerbil. J Stroke Cerebrovasc Dis.

[54] Sui L, Nomura R, Dong Y, Yamaguchi F, Izumori K, Tokuda M (2007). Cryoprotective effects of d-allose on mammalian cells. Cryobiology.

[55] Kimura S, Zhang G-X, Nishiyama A, Nagai Y, Nakagawa T, Miyanaka H, Fujisawa Y, Miyatake A, Nagai T, Tokuda M, Abe Y (2005). D-allose, an all-cis aldo-hexose, suppresses development of salt-induced Hypertension in Dahl rats. J Hypertens.

[56] Hossain MA, Wakabayashi H, Goda F, Kobayashi S, Maeba T, Maeta H (2000). Effect of the immunosuppressants FK506 and D-allose on allogenic orthotopic liver transplantation in rats. Transplantation Proceedings.

[57] Ishihara Y, Katayama K, Sakabe M, Kitamura M, Aizawa M, Takara M, Itoh K (2011). Antioxidant properties of rare sugar D-allose: effects on mitochondrial reactive oxygen species production in Neuro2A cells. J Biosci Bioeng.

[58] Zhang M, Fu YH, Luo YW, Gou MR, Zhang L, Fei Z, Gao DK (2023). d-allose protects brain microvascular endothelial cells from hypoxic/reoxygenated injury by inhibiting endoplasmic reticulum stress. Neurosci Lett.

[59] Khajeh S, Ganjavi M, Panahi G, Zare M, Zare M, Tahami SM, Razban V. D-allose: molecular pathways and therapeutic capacity in cancer. Curr Mol Pharmacol. 2023;16(8):801–10. 10.2174/1874467216666221227105011 36578261

[60] Kanaji N, Kamitori K, Hossain A, Noguchi C, Katagi A, Kadowaki N, Tokuda M (2018). Additive antitumour effect of D–allose in combination with cisplatin in non-small cell Lung cancer cells. Oncol Rep.

[61] Hossain MA, Izuishi K, Maeta H (2003). Protective effects of D-allose against ischemia reperfusion injury of the rat liver. J Hepatobiliary Pancreat Surg.

[62] Mizote M, Hirooka K, Fukuda K, Nakamura T, Itano T, Shiraga F (2011). D-allose as ischemic retina injury inhibitor during rabbit vitrectomy. Jpn J Ophthalmol.

[63] Ju J, Hou R, Zhang P (2020). D-allose alleviates ischemia/reperfusion (I/R) injury in skin flap via MKP-1. Mol Med.

[64] Le Mercier M, Fortin S, Mathieu V, Kiss R, Lefranc F (2010). Galectins and gliomas. Brain Pathol.

[65] Doverhag C, Hedtjarn M, Poirier F, Mallard C, Hagberg H, Karlsson A, Savman K (2010). Galectin-3 contributes to neonatal hypoxic-ischemic brain injury. Neurobiol Dis.

[66] Lin CI, Whang EE, Donner DB, Jiang X, Price BD, Carothers AM, Delaine T, Leffler H, Nilsson UJ, Nose V (2009). Galectin-3 targeted therapy with a small molecule inhibitor activates apoptosis and enhances both chemosensitivity and radiosensitivity in papillary thyroid cancer. Mol Cancer Res.

[67] Arumugam TV, Okun E, Tang SC, Thundyil J, Taylor SM, Woodruff TM (2009). Toll-like receptors in ischemia-reperfusion injury. Shock.

[68] Xu GR, Zhang C, Yang HX, Sun JH, Zhang Y, Yao TT, Li Y, Ruan L, An R, Li AY (2020). Modified citrus pectin ameliorates myocardial fibrosis and inflammation via suppressing galectin-3 and TLR4/MyD88/NF-kappaB signaling pathway. Biomed Pharmacother.

[69] Feng C, Wan H, Zhang Y, Yu L, Shao C, He Y, Wan H, Jin W (2020). Neuroprotective effect of Danhong Injection on Cerebral Ischemia-Reperfusion Injury in rats by activation of the PI3K-Akt pathway. Front Pharmacol.

[70] Li D, Guo YY, Cen XF, Qiu HL, Chen S, Zeng XF, Zeng Q, Xu M, Tang QZ (2022). Lupeol protects against cardiac hypertrophy via TLR4-PI3K-Akt-NF-kappaB pathways. Acta Pharmacol Sin.

